# Laboratory-based surveillance of current antimicrobial resistance patterns and trends among *Staphylococcus aureus*: 2005 status in the United States

**DOI:** 10.1186/1476-0711-5-2

**Published:** 2006-02-09

**Authors:** David Styers, Daniel J Sheehan, Patricia Hogan, Daniel F Sahm

**Affiliations:** 1 Focus Bio-Inova, Inc., 13665 Dulles Technology Drive, Herndon, VA 20171, USA; 2 Pfizer, Inc, Pfizer Global Pharmaceuticals, 234 E. 42nd Street, New York, N.Y. 10017, USA

## Abstract

**Background:**

The virulence, antimicrobial resistance, and prevalence of *S. aureus *underscores the need for up-to-date and extensive insights regarding antimicrobial susceptibility trends. One approach to meet this need is analysis of clinical laboratory – based surveillance data.

**Methods:**

Data from The Surveillance Network-USA (TSN), an electronic surveillance network that collects microbiology data from 300 clinical microbiology laboratories across the United States, were used as the source for analysis that included prevalence of *S. aureus *in clinical specimens, MRSA and multi-drug resistance phenotype rates and trends according to patient location, geographic distributions, and specimen source.

**Results:**

*S. aureus *was the most prevalent species isolated from inpatient specimens (18.7% of all bacterial isolates) and the second most prevalent (14.7%) from outpatient specimens. In March 2005 MRSA rates were 59.2%, 55%, and 47.9% for strains from non-ICU inpatients, ICU, and outpatients, respectively. This trend was noted in all nine US Bureau of Census regions and multi-drug resistance phenotypes (resistance to ≥ 3 non-beta-lactams) was common among both inpatient MRSA (59.9%) and outpatient MRSA (40.8%). Greater than 90% of multi-drug resistant MRSA were susceptible to trimethoprim-sulfamethoxazole, linezolid, and vancomycin.

**Conclusion:**

Prevalence of MRSA among both inpatient and outpatient specimens continues to increase with multi-drug resistance as a common phenotype. Continued emergence of outpatient MRSA that exhibit multi-drug resistant phenotypes has important implications for developing and evolving outpatient treatment guidelines.

## Background

*Staphylococcus aureus *exhibits three problematic features that, taken together, are not found among most other clinically relevant bacteria. This species is capable of expressing a variety of virulence factors and thus is almost always considered medically relevant when encountered in clinical specimens; the organism continues to demonstrate the ability to develop and expand resistance to include a broad array of antimicrobial classes, and *S. aureus *is a prominent pathogen in both the hospital and the community settings [1,2]. This combination of characteristics underscores the need to monitor and report *S. aureus *trends and patterns in a timely and thorough manner, especially with regard to antimicrobial susceptibility profiles and the clinical settings in which the organism is encountered.

To gain more extensive insights regarding recent antimicrobial trends among *S. aureus*, analysis of strain data obtained from geographically and demographically diverse patient populations in the United States is necessary. One approach that can provide key information in this regard involves the application of laboratory – based surveillance. Analysis of microbiology data generated in support of patient care by clinical laboratories across the United States has been used in the past to provide perspective on a variety of antimicrobial resistance trends among key bacterial pathogens [3-6]. Further, Fridkin et al [7] have reported that laboratory derived antimicrobial data can provide reliable perspectives on resistance rates among patients with hospital – acquired infections. Therefore, to help meet the need for current and broad-based information regarding *S. aureus *trends, data obtained through The Surveillance Network-USA (TSN) were analyzed for resistance trends overall and in both the inpatient and outpatient settings.

## Methods

The Surveillance Network (TSN) was the data source used for this investigation and analysis. TSN is an electronic database of strain specific, qualitative and quantitative antimicrobial susceptibility test data reported by clinical laboratories in North America that has been used extensively in the past to evaluate various trends regarding antimicrobial susceptibility [3-6]. At the time of this analysis TSN contained more than 105 million susceptibility test results overall that were gathered from 300 USA hospitals distributed across the United States. In addition to antimicrobial susceptibility profiles other query parameters that may be used individually or in any combination for analysis of antimicrobial susceptibility data include organism identification, national and regional geography (i.e. the nine regions of the US Census Bureau), institution demographics (type, number of beds), patient demographics (age, gender, and location), and specimen source.

This study focused specifically on *S. aureus *and used TSN data collected from 1998 to March 2005. The overall prevalence of *S. aureus *isolated from inpatient and outpatient specimens was calculated by using all organism groups and species isolated from each patient group as the denominator. MRSA rates for the study period were examined overall and according to three patient location categories as designated by each laboratory's information system (outpatient, inpatient [non-ICU], and ICU). The outpatient designation indicates that the specimen submitted for culture was obtained from patients seen in an outpatient setting. For all patient locations three categories of clinical specimen source were analyzed and included lower respiratory tract, skin and soft tissue (included wounds), and blood. Multi-drug resistance for MRSA was defined as resistance to three or more agents among ciprofloxacin, erythromycin, clindamycin, gentamicin, trimethoprim/sulfamethoxazole, linezolid, and vancomycin. Only strains tested simultaneously against all of these agents were included in the multi-drug resistance analysis of prevalence and distribution of resistance phenotypes.

## Results

**Figure 1:**
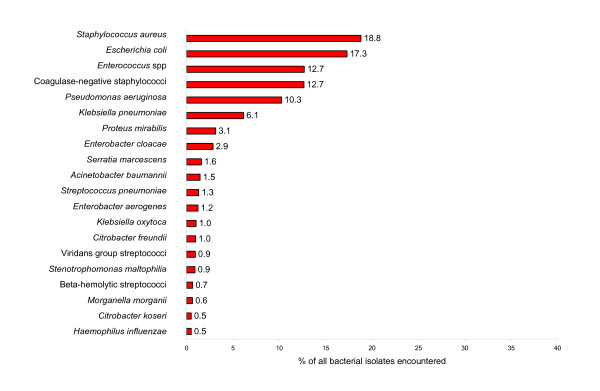
**Relative frequency of bacterial species/groups encountered in clinical specimens from inpatients**. Data is cumulative data: 1998 – March 2005 and based on a total of 3,209,413 bacterial isolates.

**Figure 2:**
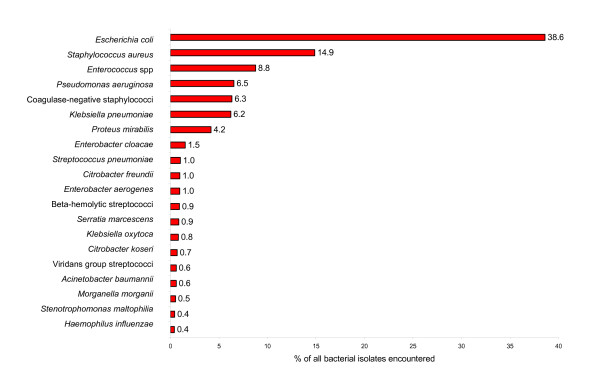
**Relative frequency of bacterial species/groups encountered in clinical specimens from outpatients**. Data is cumulative data: 1998 – March 2005 and based on a total of 3,209,413 bacterial isolates.

The top 20 most commonly reported bacterial species or organism groups isolated from inpatient and outpatients, as reported by clinical laboratories throughout the United States from 1998 to 2005, are shown in Figures 1 and 2, respectively. For both inpatient and outpatient specimens, *S. aureus *and *E. coli *were the two most common species reported. Among inpatients, *S. aureus *was more prominent (18.7%) than *E. coli *(17.3%). For outpatient specimens the reverse was noted; *E. coli *(38.6%) and *S. aureus *(14.7%). The relative incidence of *S. aureus *among all reported organisms was similar for inpatients (18.7%) and outpatients (14.7%).

**Figure 3:**
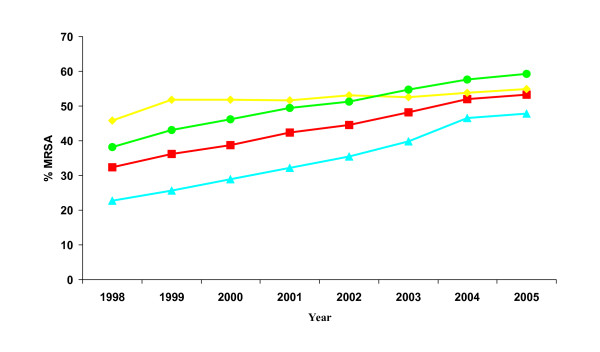
**MRSA trends (1998 – YTD 2005) according to patient location**. Data is cumulative data: 1998 – March 2005. Red line, all patients; yellow line, ICU patients; green line, inpatients; blue line, outpatients.

With regard to resistance trends, overall MRSA rates have steadily increased in the USA since 1998 and the rate appeared to be still on the rise as of March, 2005 (53.3%: Figure 3). An increase was noted among each patient group including strains from ICU patients, non-ICU inpatients, and outpatients with current MRSA rates of 55%, 59.2%, and 47.9%, respectively. Since 2002 the lowest rate of increase in MRSA occurred among specimens from ICU patients while the rates among *S. aureus *from other inpatients and outpatients were higher.

**Figure 4:**
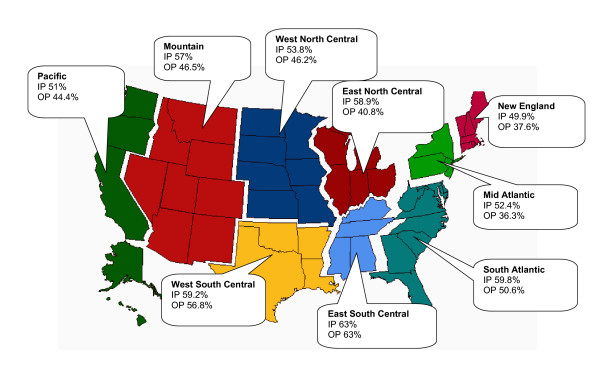
**Inpatient (IP) and outpatient (OP) MRSA rates according to US Census Bureau Regions**. Data is cumulative data: 1998 – March 2005.

Analysis of inpatient and outpatient MRSA rates according to geographic distribution demonstrated that trends for MRSA have occurred in each of the nine regions of the US Census Bureau (Figure 4). In all regions, except New England, inpatient MRSA rates were above 50%. The lowest outpatient MRSA rates occurred in the Mid Atlantic (36.3%) and New England (37.6%) regions; while the highest rate (63%) occurred in the East South Central region where inpatient and outpatient MRSA rates were the same.

According to specimen source, MRSA rates were highest (55.9%) among strains from inpatient lower respiratory specimens and lowest (37.6%) among strains from outpatient skin and soft tissue specimens (Table 1). For both inpatients and outpatients the range of MRSA rates according to specimen source was relatively narrow, 48.6 – 55.9% and 37.6 – 42.8%, respectively. Approximately 41% of *S. aureus *strains obtained from 31,886 blood cultures taken in the outpatient setting were MRSA.

**Table 1:** MRSA rates among inpatients and outpatients by specimen source^a^

	Inpatient		Outpatient	
		
Specimen Source	Total N	(%) MRSA	Total N	(%) MRSA
Lower Respiratory Tract	188,939	(55.9)	51,057	(42.8)
Skin and Soft Tissue	61,099	(48.6)	56,830	(37.6)
Blood	92,694	(49.1)	31,886	(41.4)

^a^Cumulative data 1998 – March 2005

Regardless of the specimen source, the multi-drug resistance rates among MRSA were higher among inpatient strains than among outpatient strains (Table 2). For both inpatient and outpatient MRSA, the highest multi-drug resistance rates occurred among lower respiratory tract isolates (67.8% for inpatients; 65% for outpatients) and the lowest rates were among skin and soft tissue specimens (35.8% for inpatients; 22.2% for outpatients).

**Table 2:** Multi-drug resistance (MDR)^a ^rates among MRSA from inpatients and outpatients by specimen source^b^

	Inpatient		Outpatient	
		
Specimen Source	Total N	(%) MDR	Total N	(%) MDR
Lower Respiratory Tract	5,134	(67.8)	1,136	(65.0)
Skin and Soft Tissue	2,122	(35.8)	2,275	(22.2)
Blood	3,064	(63.3)	904	(56.7)

^a^Multiple-drug resistance (MDR): resistance to three or more of the following agents: ciprofloxacin, erythromycin, clindamycin, gentamicin, trimethoprim/sulfamethoxazole, linezolid, and vancomycin

^b^Cumulative data 1998 – March 2005

For outpatient and inpatient MRSA combined, 29 different resistance phenotypes were noted, 24 of which occurred in both populations (Table 3). The spectrum of phenotypes ranged from susceptibility to all non-beta-lactams to resistance to five of the seven non-beta-lactams included in the multi-drug resistance analysis. Resistance to vancomycin was not encountered and non-susceptibility to linezolid occurred with three of 14,635 MRSA strains (0.02%) profiled in this analysis. Susceptibility to all non-beta-lactams studied was more common among outpatient MRSA (5.7%) than among inpatient MRSA (4.1%), but the frequencies of the susceptible phenotype were comparable between MRSA strains from these two patient populations.

**Table 3:** Distribution of resistance phenotypes^a ^among inpatient and outpatient MRSA^b^

		Inpatient		Outpatient	
		
Category	Resistance phenotype	n	(%)	n	(%)
Susceptible to all other agents	-	418	(4.1)	245	(5.7)
					
Single-drug resistant	Eryth	1409	(13.7)	1200	(27.8)
	Cipro	232	(2.2)	92	(2.1)
	Gent	8	(0.1)	3	(0.1)
	Clinda	4	(0.0)^c^	5	(0.1)
					
Double-drug resistant	Cipro, Eryth	1854	(18.0)	870	(20.2)
	Eryth, Clinda	114	(1.1)	104	(2.4)
	Cipro, Clinda	62	(0.6)	23	(0.5)
	Cipro, Gent	8	(0.1)	12	(0.3)
	Cipro, SXT	14	(0.1)	2	(0.0)^c^
	Eryth, SXT	2	(0.0)^c^	1	(0.0)^c^
	Eryth, Gent	10	(0.1)	1	(0.0)^c^
	Gent, SXT	2	(0.0)^c^	0	(0.0)
					
Multidrug-resistant	Cipro, Eryth, Clinda	4915	(47.6)	1417	(32.8)
	Cipro, Eryth, Gent	30	(0.3)	18	(0.4)
	Cipro, Eryth, SXT	23	(0.2)	7	(0.2)
	Cipro, Gent, SXT	18	(0.2)	10	(0.2)
	Eryth, Clinda, Gent	5	(0.0)	5	(0.1)
	Cipro, Clinda, Gent	6	(0.1)	3	(0.1)
	Eryth, Clinda, SXT	2	(0.0)^c^	1	(0.0)^c^
	Eryth, Gent, SXT	0	(0.0)	1	(0.0)^c^
	Cipro, Clinda, SXT	2	(0.0)^c^	0	(0.0)
	Cipro, Eryth, Lin	3	(0.0)^c^	0	(0.0)
	Cipro, Eryth, Clinda, Gent	858	(8.3)	214	(5.0)
	Cipro, Eryth, Clinda, SXT	58	(0.6)	14	(0.3)
	Cipro, Eryth, Gent, SXT	12	(0.1)	4	(0.1)
	Eryth, Clinda, Gent, SXT	2	(0.0)^c^	0	(0.0)
	Cipro, Clinda, Gent, SXT	2	(0.0)^c^	0	(0.0)
	Cipro, Eryth, Clinda, Gent, SXT	247	(2.4)	63	(1.5)
	Total n	10,320		4,315	

^a^Analysis included the following agents: gentamicin (Gent), erythromycin (Eryth), clindamycin (Clinda), trimethoprim-sulfamethoxazole (SXT), ciprofloxacin (Cipro), vancomycin (Vanc), and linezolid (Lin). Multi-drug resistance included resistance to three or more of the agents listed.

^b^Cumulative data 2002 – March 2005

^c^n < 0.1% of total

The most common resistance phenotypes among inpatient MRSA were multi-drug resistance to ciprofloxacin, erythromycin, and clindamycin (47.6%), double drug resistance to ciprofloxacin and erythromycin (18%), single drug resistance to erythromycin (13.7%), and multi-drug resistance to ciprofloxacin, erythromycin, clindamycin, and gentamicin (8.3%) (Table 3). Among outpatient MRSA single drug resistance to erythromycin (27.8%) was the most common phenotype followed by multi-drug resistance to ciprofloxacin, erythromycin, and clindamycin (32.8%), and dual resistance to ciprofloxacin and erythromycin (20.2%). In all, 11.4% (n = 1,179) of inpatient MRSA were resistant to four or more non-beta-lactam agents and 2.4% (n = 247) were resistant to five agents. In comparison, 6.8% (n = 295) of outpatient MRSA were resistant to four or more non-beta-lactams and 1.5% (n = 63) were resistant to five agents.

For both outpatient and inpatient multi-drug resistant MRSA the only agents with > 90% susceptibility were trimethoprim/sulfamethoxazole, linezolid, and vancomycin (Table 4). This same hierarchy of antimicrobial activity was maintained when data were analyzed according to clinical specimen source (data not shown). The > 99.9% linezolid susceptibility for inpatient MRSA strains resulted from three of 6,183 strains being reported as non-susceptible.

**Table 4:** Antibiograms of multi-drug resistant^a ^MRSA from inpatients and outpatients^b^

	Inpatient (n = 6,183)		Outpatient (n = 1,757)	
		
Agents	n	(%) Susceptible	n	(%) Susceptible
Ciprofloxacin	7	(0.1)	5	(0.3)
Erythromycin	20	(0.3)	13	(0.7)
Clindamycin	85	(1.4)	38	(2.2)
Gentamicin	4,971	(80.4)	1,430	(81.4)
Trimethoprim-sulfamethoxazole	5,814	(94.0)	1,657	(94.3)
Linezolid	6,180	(>99.9)	1,757	(100)
Vancomycin	6,183	(100.0)	1,757	(100)

^a^Multiple-drug resistance: resistance to three or more of the following agents: ciprofloxacin, erythromycin, clindamycin, gentamicin, trimethoprim/sulfamethoxazole, linezolid, and vancomycin

^b^Cumulative data 1998 – 2005 YTD

## Discussion and conclusion

This analysis of data obtained through clinical laboratory based surveillance has demonstrated that *S. aureus *continues to be solidly positioned as a leading bacterial pathogen encountered in both inpatient and outpatient clinical settings and has continued to substantially outdistance other gram-positive organisms in this regard. Because most clinical microbiology protocols call for *S. aureus *to be "worked-up" and reported regardless of the amount of organisms present, or the type of specimen, there is a likelihood that the reporting frequency based on laboratory data is higher than the actual frequency of infections caused by this organism. Nonetheless, the same bias plays a role for many other reported bacterial species so that the position of *S. aureus *as the leading gram-positive organism, and its frequency relative to other species, is likely quite reflective of actual infection rates as well. The high prevalence of *S. aureus *reported here for both the inpatient and outpatient environments was consistent with recent descriptions and discussion regarding the changing epidemiology of this organism [1,2].

The high prevalence of *S. aureus *in both settings underscores the need for analysis of antimicrobial resistance trends according to patient location so that variations in antimicrobial resistance trends among strains from patients seen in the different clinical settings can be evaluated. Although previous surveillance initiatives that included *S. aureus *have involved isolates from both outpatients and inpatients, they examined strains collected prior to 2002 and a thorough comparison of the resistance rates and phenotypes according to patient location were not reported [8,9]. In this current study antimicrobial data from geographically, demographically, and clinically diverse settings, collected via The Surveillance Network, were analyzed to provide a clearer and current perspective on the resistance profiles currently present among *S. aureus *in the United States.

The data provided in Figure 3 demonstrated that MRSA rates continue to be on the rise among both inpatients and outpatients, and this trend appeared to be pervasive throughout all regions of the United States (Figure 4). Rising rates of MRSA in the hospital environment were reported from studies done in previous years and data presented here indicate that the increasing rates have continued into 2005 [7,10-12]. The relatively lower increase in MRSA observed here among ICU *S. aureus *is consistent with the most recent report from the National Nosocomial Infections Surveillance (NNIS) System Report [12]. Although the rate increase may be relatively lower than before, the overall prevalence of MRSA in the ICU remains high (55%).

In addition to the issue of increasing MRSA rates among inpatients, the recognized emergence of MRSA beyond the hospital or healthcare setting into the community has raised another substantial public health concern about *S. aureus *[2,13-17]. Based on this current laboratory – based surveillance study the concern is much warranted as outpatient MRSA rates have continued to increase to the extent that as of 2005 the overall MRSA rate in this population was 47.9% (Figure 3). Further, the data in Table 1 indicated that MRSA are commonly isolated from all types of outpatient specimens, including blood.

MRSA that are encountered in the community (outpatient settings) arise either as a result of acquisition of the *mec *gene complex by susceptible *S. aureus *strains (so-called *de novo *community MRSA), or by person-to-person carriage of hospital strains into the community [2,18]. No phenotypic or genotypic criteria have been firmly established to definitively specify a strain as being from one or the other origin; this requires rigorous and diligent epidemiological analysis. However, *de novo *MRSA have generally been characterized as being susceptible to most non-beta-lactam agents (other than erythromycin) while those that have spread from the hospital setting into the community often exhibit the multi-drug resistant phenotype that is characteristic of most hospital-based strains [2,13,18,19].

The significant contrast in MRSA resistance phenotypes that may exist depending on the origin of a community strain (i.e. hospital or *de novo*) can confound empiric therapeutic decisions when patients suspected of being infected with *S. aureus *are initially evaluated in the outpatient setting. While the rates of multi-drug resistant phenotypes among inpatient MRSA were higher than those among outpatient MRSA, multi-drug resistance was a common feature among the community outpatient population (Table 2). In addition, there was substantial overlap in phenotypes between inpatient and outpatient MRSA (Table 3). These findings, based on data from across the USA collected since 1998, indicate that strains of hospital origin constitute the majority of MRSA encountered in the outpatient setting. This is consistent with the local findings reported by Charlebois et al. [18] in which a substantial proportion of outpatient MRSA had resistance profiles comparable to those of the hospital-based strains. Their analysis led the authors to suggest that most of the community MRSA strains from the area studied around San Francisco were likely descendants of hospital origin. Further, a previous study we conducted also strongly suggested that community MRSA frequently emerge from the local hospital populations [4].

For multi-drug resistant inpatient and outpatient MRSA alike there are relatively few agents that maintained high levels of activity (Table 4). This situation combined with the propensity of certain community clones to exhibit substantial virulence with elaboration of toxins such as Panton-Valentine Leuckocidin (PVL) raises concern about future therapeutic guidelines for *S. aureus *infections encountered in the outpatient settings [20-22].

The most common multi-drug resistant phenotype encountered would preclude the use of all current beta-lactams, clindamycin, macrolides, and fluoroquinolones against outpatient MRSA. Although clindamycin is frequently considered for infections in this setting, over 30% of outpatient MRSA were resistant. The agents that consistently demonstrated high levels of activity against outpatient MRSA, including strains exhibiting the multi-drug resistant phenotype, were trimethoprim/sulfamethoxazole, linezolid, and vancomycin. No resistance to either vancomycin or linezolid was encountered among outpatient MRSA, including multi-drug resistant strains. Therefore, while there have been reports of resistance to these two agents, resistance remains sporadic and extremely rare [1,23]. Because resistance to either vancomycin or linezolid is extremely rare among all *S. aureus*, use of either agent is not likely to lead to expansion of multi-drug resistant clones as could result from use of the other agents to which *S. aureus *populations have already developed resistance. However, with regard to vancomycin, the lack of an oral formulation with absorption introduces a limitation as an option for the management of outpatient MRSA infections. Linezolid has exhibited successful activity relative to vancomycin for MRSA and is available as an oral formulation [24-26].

The continued increase in MRSA rates among inpatient specimens coupled with the emergence of MRSA in the community (outpatient) setting has involved strains that frequently exhibit multiple drug resistance. For some time these resistance phenotypes have been an issue for the management of inpatients. Now current trends indicate there are important implications for establishing outpatient management and treatment guidelines for staphylococcal infections. Given this trend, health care institutions should consider analyzing their local *S. aureus *antibiograms according to outpatient and inpatient populations in order to discern the prevalent phenotypes physicians are likely to encounter in each setting. Finally, if the current trend continues, the development of new anti-MRSA agents for multi-drug resistant strains will have to consider the need for both community and hospital use.

## Authors' contributions

Conception and design: P Hogan, DJ Sheehan, DF Sahm.

Assembly of data and analysis: D Styers, DF Sahm.

Drafting of the article: P Hogan, DJ Sheehan, DF Sahm.
